# Rapid colorimetric sensing of chlorpromazine HCl antipsychotic through *in situ* growth of gold nanoparticles†

**DOI:** 10.1039/d3ra05516g

**Published:** 2024-01-11

**Authors:** Mohammad K. Hammood, Jalal N. Jeber, Maryam A. Khalaf, Haneen Abdul hadi kharaba

**Affiliations:** a Department of Chemistry, College of Science, University of Baghdad 10071 Baghdad Iraq jalal.n@sc.uobaghdad.edu.iq +9647702519630; b Ministry of Education Karkh Education Directorate 1 Baghdad Iraq; c Department of Pharmaceutics, College of Pharmacy, University of Baghdad Baghdad Iraq

## Abstract

Antipsychotic drugs like chlorpromazine hydrochloride (CPZ) are widely used to treat mental illnesses but can accumulate in the environment if not properly disposed of. Long-term exposure to trace levels of such pharmaceuticals may pose health risks. This study reports a colorimetric assay for detection of the antipsychotic drug chlorpromazine hydrochloride (CPZ) based on its ability to reduce gold ions and form gold nanoparticles (AuNPs). Optimization of reaction conditions such as pH, temperature and reagent concentrations enabled quantitative analysis of CPZ concentrations from 0.1–30 μg mL^−1^, with a detection limit of 0.06 μg mL^−1^, 0.23 μg mL^−1^ quantification limit and less than 3.5% RSD. The AuNPs exhibited a characteristic surface plasmon resonance band at 527 nm detectable by UV-vis spectrophotometry. Method validation with spiked serum, urine and environmental water samples demonstrated acceptable accuracy and precision. Interfering substances showed minimal impact, indicating resilience and specificity. This rapid, inexpensive colorimetric assay could facilitate environmental monitoring and biomedical analysis of antipsychotic drugs.

## Abstract 2

This assay rapidly detects chlorpromazine hydrochloride using its ability to reduce gold ions to form nanoparticles. Its low cost, resilience to interferences and short analysis time could facilitate environmental monitoring and biomedical analysis.

## Graphical abstract

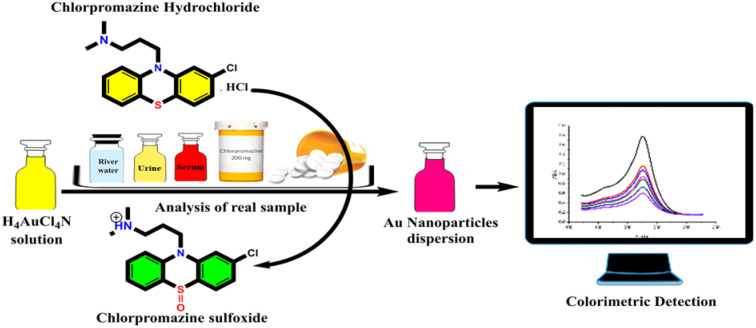

## 1 Introduction

Gold nanoparticles (AuNPs) have unique optical and chemical properties that make them well-suited for analytical sensing applications. When AuNPs have dimensions smaller than 50 nm, they exhibit strong light absorption in the visible region due to surface plasmon resonance.^1–3^ The position and intensity of this absorption peak depends on factors like AuNP size, shape and aggregation state.^4,5^ Importantly, the surface plasmon resonance absorption of AuNPs allows for highly sensitive and quantitative measurement of their concentration using simple spectrophotometry. Researchers have leveraged this property to develop colorimetric biosensors using AuNPs modified with biorecognition elements like aptamers, these optical sensors have enabled detection of targets such as metal ions,^6^ protease activity,^7^ and protein^8^ at trace levels. Aggregation-induced changes in AuNP plasmon resonance provide the basis for many “lab-on-a-particle” sensing platforms. For example, engineered aggregation through DNA hybridization or nanoparticle conjugation has enabled protein analysis, enzyme activity assays, ion detection and DNA sequencing applications.^9^ Early work in this area by Willner *et al.* demonstrated that neurotransmitters acting as *in situ* reducing agents could enable the generation of AuNPs, whose optical properties allow quantitative analysis of different neurotransmitters.^10^ This approach has also been applied to detect other analytes like H_2_O_2_ (ref. 11) and sugars,^12^ and antioxidant activity^13^ using AuNP-based colorimetric assays. The determination of drugs is of utmost importance for numerous reasons. Accurate drug determination ensures proper dosage administration, helps monitor drug efficacy, assesses therapeutic drug levels, and aids in detecting drug abuse or misuse. It plays a vital role in patient safety, pharmacological research, and the overall management of healthcare. Therefore, a series of methods have been developed for the determination of various drugs, including flow injection,^14–16^ turbidity,^17–19^ solid phase extraction,^20^ HPLC,^21^ electrochemistry^22^ and spectrophotometry.^23,24^

Inspired by these revolutionary works, a team of proficient scientists and researchers has successfully developed a colorimetric assay using AuNPs, enabling the swift detection of β-agonists. This assay specifically focuses on phenylethanolamine compounds with various substituent groups on the aromatic ring as its primary targets.^25^ The resultant colorimetric assay may prove to be a versatile method for detecting a range of reductive analytes, and to evaluate this possibility, the team selects CPZ antipsychotic as a model system.

CPZ, a phenothiazine therapeutic agent, possesses, sedative, analgesic, anticholinergic, antipsychotic, and antihistaminic properties, and is predominantly utilized for the treatment of allergic symptoms, motion sickness prevention, and anxiety.^26^ However, caution must be exercised during CPZ administration due to the potential occurrence of severe side effects in humans, such as drowsiness, dry mouth, endocrine disorders, blurred vision, occasional hypotension, respiratory depression, nausea, constipation, akathisia, dystonia and trouble sleeping. Precise CPZ detection is crucial in biological samples and pharmaceutical preparations and due to the gravity of its adverse effects.^27^ The ubiquity of CPZ usage for therapeutic purposes, sometimes concurrently with other medications, has led to its detection in aquatic environments. Consequently, CPZ presence in water systems is a matter of increasing environmental concern. The Globally Harmonized System of Classification and Labelling of Chemicals (GHS) recognizes CPZ as hazardous to the aquatic realm due to its acute and lingering toxic effects on aquatic organisms.^28,29^ As CPZ is so widely employed clinically, yet its potentially adverse impacts on aquatic life are acknowledged, development of analytical techniques for CPZ monitoring in water becomes increasingly important from environmental stewardship perspectives.

Numerous analytical techniques have been employed for the detection of CPZ, including capillary zone electrophoresis,^30^ GC/MS,^31^ HPLC,^32^ and chemiluminescence technique.^33^ Furthermore, in recent times, a host of biosensors and electrochemical sensors have been developed and used to identify various target analytes related to food quality, environmental pollutants, biomarkers, pesticides, and pharmaceuticals. To achieve optimal selectivity and sensitivity, bare electrodes are customized with functional nanomaterials, thereby influencing the overall performance of the sensors. With the progression of nanotechnology, functional nanomaterials like metal nanoparticles (NPs), polymeric nanomaterials, transition metal oxides, nano carbmetal chalcogenide and their composites are employed as electrode materials.^34^ Although these methods offer high sensitivity and reliability when quantifying CPZ in commercial pharmaceutical preparations, most of them require long time analysis, complicated procedures, professional training, and costly instrumentation, which renders those conventional methods unsuitable for real-time and on-site detection. Consequently, a colorimetric technique has emerged as a favourable alternative due to its advantages like rapid analysis, operational simplicity, high sensitivity, field applicability, and cost-effectiveness.

In this study, a facile and expeditious colorimetric approach has been devised for the determination of CPZ in liquid samples (urine and serum) as well as CPZ tablet and river water samples, involving *in situ* growth of gold nanoparticles (AuNPs). Upon combining CPZ with ammonium chloroaurate (NH_4_AuCl_4_) within the solution, a distinctive red color attributed to the SPR band of AuNPs is instantaneously observed at ambient temperature. The absorbance measured at 527 nm is linearly proportional to the concentration of CPZ present in the analyzed solution. Thus, the developed colorimetric assay can be effectively employed for rapid, *in situ* screening of CPZ in liquid samples, requiring minimal sample preparation. As a result, this work holds significance in the field of analytical chemistry and has implications for the effective monitoring and regulation of CPZ in environmental and industrial settings.

## 2 Experiment

### 2.1 Materials and apparatus

Sigma (St. Louis, MO) provided chlorpromazine hydrochloride, sodium chloride, ammonium chloroaurate salts, boric acid, sodium tetraborate, ethanol, methanol, urea, uric acid, glucose, creatinine and trichloroacetic acid (TCA), which all were of analytical grade, >99.9%. Aqueous solutions were prepared using ultrapure water (Milli-Q, Millipore Corporation, Bedford, MA). The UV-L9, UV-visible spectrophotometer (WEST TUNE, China) equipped with glass cuvettes (1.0 cm) was employed to conduct spectrophotometric measurements, which were performed at wavelengths between 200 and 800 nm. The Field Emission Scanning Electron Microscopy (FE-SEM) images of AuNPs were obtained from a Teneo Field Emission Scanning Electron Microscopy (Thermo Fisher Scientific, USA) using an acceleration voltage of 500 V to 30 kV. FE-SEM samples were prepared by placing 2.0 mL solution containing 20 μg mL^−1^ of AuNPs onto a glass plate and drying in air. The synthesized gold nanoparticles were observed using a JEM-2010 transmission electron microscope (TEM). Energy Dispersive X-ray spectroscopy (Type EDX 8100, Shimadzu, Japan) was used to determine the composition of samples. The morphology and physical properties of AuNPs obtained were investigated using an AFM NEXT II (NT-MDT). The particle size analysis from AFM, TEM and FE-SEM images was carried out using the ImageJ software, an image processing program. The methodologies involving human samples were approved by the Institutional Ethics Committee of [University of Baghdad/College of Science]. Written informed consent was obtained from all participants prior to collection of blood and urine samples. All experiments were performed in accordance with relevant guidelines and regulations regarding the use of human specimens for biomedical research. Appropriate precautions were taken during sample collection, handling, analysis and disposal to protect participant privacy and safety.

### 2.2 Preparation of real samples for the colorimetric assay

The analysis of real samples involved examining various biological fluids and environmental sources that may contain CPZ residues. The human serum sample was obtained from residual specimens' leftover from routine testing procedures conducted at Al-Yarmouk Hospital in Baghdad, Iraq. These samples were screened to ensure they came from healthy individuals not taking any medications to eliminate potential interference. A mid-stream urine sample was collected from 25–35 year-old healthy male volunteers with no history of antipsychotic use in the past six months. A CPZ tablet was sampled from an intact blister strip from a commercial package purchased from a pharmacy in Central Baghdad. The river water sample was collected in pre-washed polyethylene bottles from three different locations (Babylon village 32.04°N 44.25°E, Baiji 34.52°N 43.58°E and Taji 33.55°N 44.07°E) along the Tigris River. All samples were filtered through 0.45 μm pore size membranes and stored in clean, dry test tubes at 4 °C in a refrigerator until analysis within one week. Storage at refrigerated temperature helped minimize degradation prior to conducting the colorimetric assay. This sampling strategy aimed to obtain representative real-world matrices for method validation, while also adhering to ethical sampling guidelines and avoidance of interference-causing contaminants. To analyze the biological samples, a test solution was prepared by taking a suitable quantity of each sample and subjecting it to centrifugation at a speed of 8000 revolutions per minute for 20 minutes. Then, 200 μL of TCA (10%) was added to the 100 μL of serum sample and gently mixed. After that, the mixture allowed to stand for 1 hour at 4 °C. Then, the test tubes were centrifuged at 3000 rpm for 10–15 minutes to obtain a protein precipitate. The supernatants were collected and transferred into another clean and dry test tube and stored at 4 °C for further experiments. The resulting supernatant liquid was then diluted with a 0.05 M STB solution. Subsequently, the diluted real samples were mixed with varying concentrations of CPZ solutions. In the final step, a suitable volume of each spiked sample was analyzed using the standard addition method in a 0.05 M STB solution (pH 7.0). To obtain the pharmaceutical preparation sample, CPZ tablets (25 mg per tablet, commercial brand) were finely powdered, and a measured quantity of this powder was dissolved in 100 mL of distilled water. The resulting mixture was sonicated for 20 minutes and subsequently filtered. The filtrate obtained underwent further dilution with a 0.05 M STB solution to prepare it for colorimetric analysis. Prior to analysis, river water samples were purified to eliminate potential interferents. Initial purification involved centrifugation of the samples at 5000 revolutions per minute for 30 minutes to separate solids. After centrifugation, the samples were carefully filtered to remove any particulates. A standard addition method was used where a known concentration of chlorpromazine (CPZ) was added to the purified river water sample. This permitted a colorimetric analysis and quantification of the amount of CPZ originally present in the samples.

### 2.3 General procedure

All of samples including serum, urine, tablet and river water were prepared for colorimetric analysis. Each sample (50 μL) was combined with NH_4_AuCl_4_ solution (0.5 mL of 20 μg mL^−1^) and STB solution (0.5 mL of 0.05 M). The mixtures were then vigorously mixed for 2 minutes. Various concentrations of CPZ were then added and incubated at 40 °C for another 2 minutes. This resulted in a visible color change from yellow to red, confirming the CPZ-catalyzed formation of AuNPs under the optimized reaction conditions. The intensity of the red color was quantified by spectrophotometry to determine the levels of CPZ in the original serum, urine, tablet and river water samples. The colorimetric assay served to detect and measure any CPZ present in the different sample types.

## 3 Results and discussion

### 3.1 Optimization of reaction variables for colorimetric assay

**Fig. 1:**
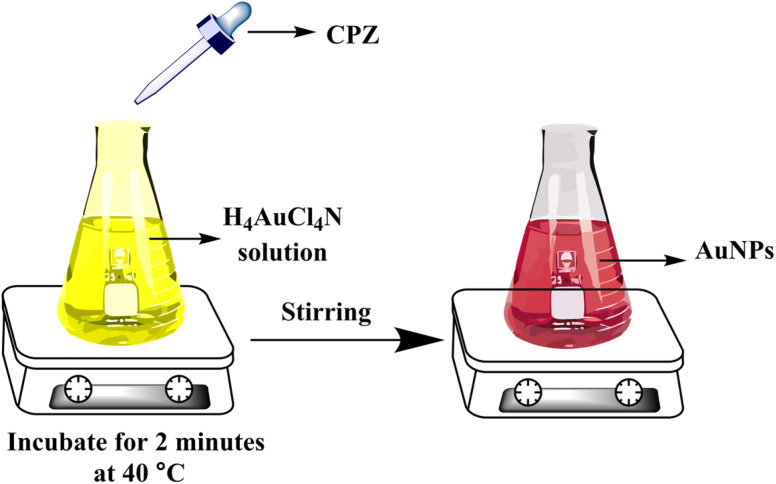
The formation step of AuNPs using the developed colorimetric assay.

**Fig. 2:**
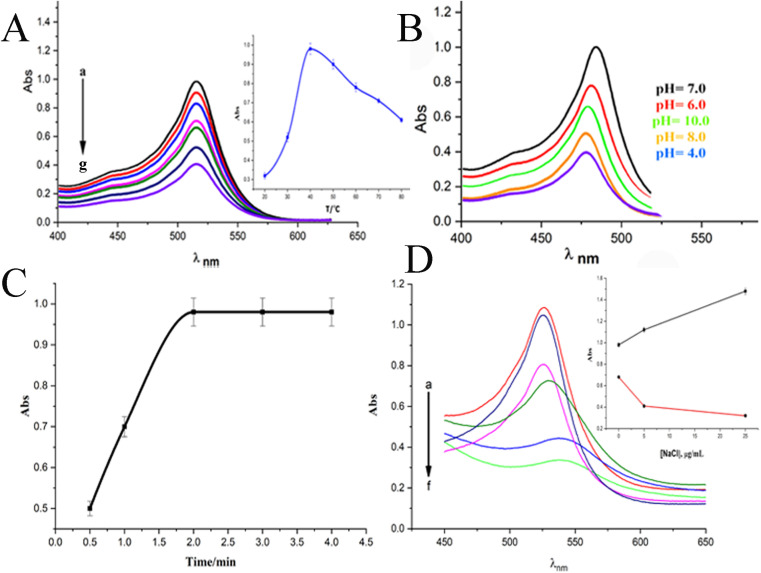
(A) Impact of incubation temperature on UV-vis spectra of formed AuNPs following the addition of 10 μg mL^−1^ of CPZ to a solution containing 20 μg mL^−1^ NH_4_AuCl_4_ and 0.05 M 0.05 M of STB buffer solution, (a) 40 °C, (b) 50 °C, (c) 60 °C, (d) 70 °C, (e) 80 °C, (f) 30 °C, (g) 20 °C. (B) Impact the pH of STB buffer solution on UV-vis spectra of formed AuNPs upon addition of 10 μg mL^−1^ of CPZ to a solution containing 20 μg mL^−1^ NH_4_AuCl_4_ and 0.05 M 0.05 M of STB at 40 °C. (C) Impact of incubation time on UV-vis spectra of formed AuNPs following the addition of 10 μg mL^−1^ of CPZ to a solution containing 20 μg mL^−1^ NH_4_AuCl_4_ and 0.05 M 0.05 M of STB buffer solution at 40 °C. (D) Impact of incubation temperature on UV-vis spectra of formed AuNPs following the addition of different concentrations of NaCl: (a) 15 μg mL^−1^, (b) 10 μg mL^−1^, (c) 5 μg mL^−1^ to a solution containing 10 μg mL^−1^ of CPZ, 20 μg mL^−1^ NH_4_AuCl_4_ and 0.05 M 0.05 M of STB buffer solution, while (d) 15 μg mL^−1^, (e) 10 μg mL^−1^, (f) 5 μg mL^−1^ of NaCl to a solution containing 4.0 μg mL^−1^ of CPZ, 20 μg mL^−1^ NH_4_AuCl_4_ and 0.05 M 0.05 M of STB buffer solution at 40 °C.

When a 10 μg mL^−1^ CPZ solution was mixed with a buffer containing 20 μg mL^−1^ NH_4_AuCl_4_, the mixture changed color visibly from yellow to red. This color change demonstrated that AuNPs formed from the reaction without requiring additional nanoparticle seeds. The spontaneous formation of AuNPs catalyzed by CPZ was observed upon the solutions being combined, as depicted in Fig. 1. The experiment parameters influencing the CPZ-catalyzed AuNP formation were then evaluated and optimized. The parameters tuned included concentrations of NH_4_AuCl_4_ and CPZ, duration of the reaction, temperature, ionic strength and pH. Fine tuning these experimental conditions ensured the growth of AuNPs proceeded efficiently in the presence of CPZ under the colorimetric assay. UV-vis spectra of the resulting AuNPs were obtained by incubating the CPZ and NH_4_AuCl_4_ mixture in a 0.05 M of STB buffer solution with a pH of 7.0 for 8 minutes at different temperatures, as presented in Fig. 2A. An intense absorbance peak at 527 nm was observed at 40 °C, and this absorbance gradually decreased between 40 °C and 70 °C. Hence, the optimal temperature for AuNP growth was determined to be 40 °C.

Neutral buffer conditions resulted in a higher absorbance at 527 nm, while acidic and basic conditions led to lower absorbance values, as illustrated in Fig. 2B. This observation can be explained by the oxidation of phenothiazine derivatives under neutral condition; the process begins with the removal of a single electron from the nitrogen atom, leading to the formation of a stable cation radical. Further elimination of another electron results in the generation of a phenothiazinium ion, followed by the subsequent formation of promethazine sulfoxide. Whereas, the released proton will be quickly neutralized by the buffer under basic condition, while the acidic buffer prevents the formation of cation radicals, both conditions do not favour the reaction. The absorbance at 527 nm was comparable to neutral pH conditions tested (pH 4.0, 6.0, 7.0, and 8.0, 10), as presented in Fig. 2B, and a pH 7.0 was chosen as the designated final pH condition.

Upon optimizing the pH and the temperature of reaction, we obtained the surface plasmon resonance (SPR) of the AuNPs from CPZ at varying incubation times (Fig. 2C). The results indicated that the absorbance reached its maximum after 2.0 minutes of incubation at 40 °C, thus determining the optimal incubation time. Following optimization, the optimal experimental parameters for the growth of AuNPs through CPZ reduction were determined as follows: incubation in a pH 7.0 buffer solution containing 20 μg mL^−1^ NH_4_AuCl_4_ at a temperature of 40 °C for a duration of 2.0 minutes.

In the pursuit of simulating biological samples like serum and urine, we embarked on a comprehensive exploration of the effects of ionic strength on buffer solutions. Fig. 2D, which showcases the SPR of AuNPs upon the addition of NaCl to the reaction mixture, was our guide. As we gradually increased the NaCl concentration (5–15 μg mL^−1^), we noticed a slight increment in absorbance when the solutions had a high concentration of CPZ (above or equal 10 μg mL^−1^, Fig. 1D). Conversely, solutions with lower concentrations of CPZ (below or equal 4.0 μg mL^−1^) exhibited a sharp decrease in absorbance as the NaCl concentration increased, alongside a red shift of the absorption peak. It's worth noting that the presence of NaCl in the reaction mixture had a significant impact on the stability of the formed AuNPs. The higher the NaCl concentrations, the greater the loss of surface charge of the AuNPs, ultimately leading to their aggregation and a decrease in absorbance at 527 nm. Hence, in the case of a 4.0 μg mL^−1^ CPZ solution, the absorbance exhibited a decreasing trend as the concentration of NaCl increased. However, for CPZ solutions with higher concentrations (equal to or above 10 μg mL^−1^), the proportion of aggregated AuNPs formed was relatively small, and the presence of NaCl in the solution had minimal impact on the absorbance of AuNPs. Based on these findings, we concluded that high ionic strength in solution samples is not suitable for CPZ detection, particularly considering the typically low concentration of CPZ in real samples.

### 3.2 Characterization of AuNPs using AFM, FE-SEM, TEM and EDX techniques

**Fig. 3:**
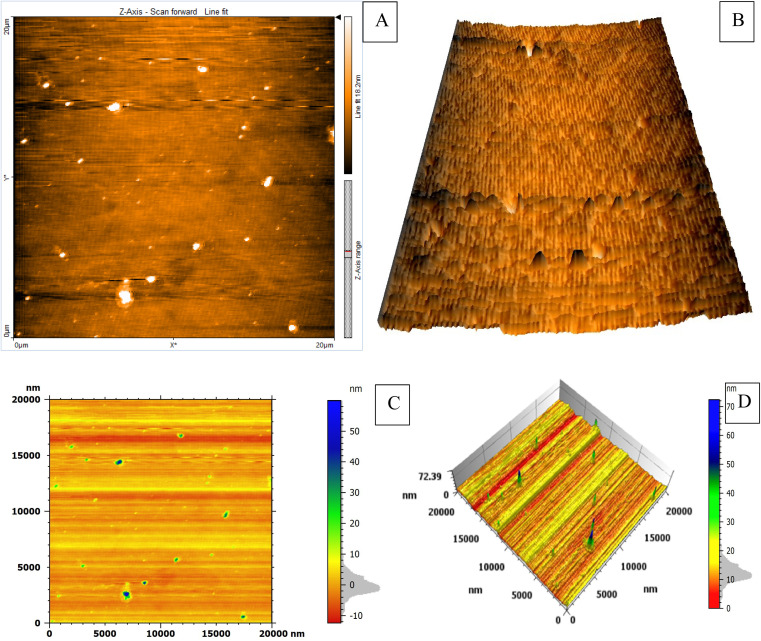
AFM micrographs of AuNPs synthesized using optimal reaction conditions. The particles were formed by adding 10 μg mL^−1^ CPZ to a solution containing 20 μg mL^−1^ NH_4_AuCl_4_ and 0.05 M sodium tetraborate buffer at 40 °C. AFM imaging visualized the AuNP morphology produced under these parameters that maximize formation catalyzed by CPZ. Image A shows the 2D surface morphology of the synthesized AuNPs. Image B shows the 3D structure of the AuNPs. Images C and D show the roughness analysis of the AuNP surface.

The study employed atomic force microscopy (AFM) to acquire high-resolution images of the AuNPs and determine their size distribution. Examination of AFM images (Fig. 3) revealed the AuNPs were uniformly distributed and exhibited a mesoporous structure, measuring 10–20 nm in size with a mean volume of 1.33 nm^3^. The size distribution was elucidated by analyzing height profiles from different areas within the AFM micrographs and computing particle diameters. The synthesized AuNP sizes resided within the typical spectrum considered appropriate for biomedical uses. Furthermore, the mesoporous construction could potentially advance intracellular incorporation and biotolerance. In brief, AFM imaging imparted important learnings by defining core properties of the AuNPs involving dimensions, dispersion throughout the sample, and porous infrastructure.

**Fig. 4:**
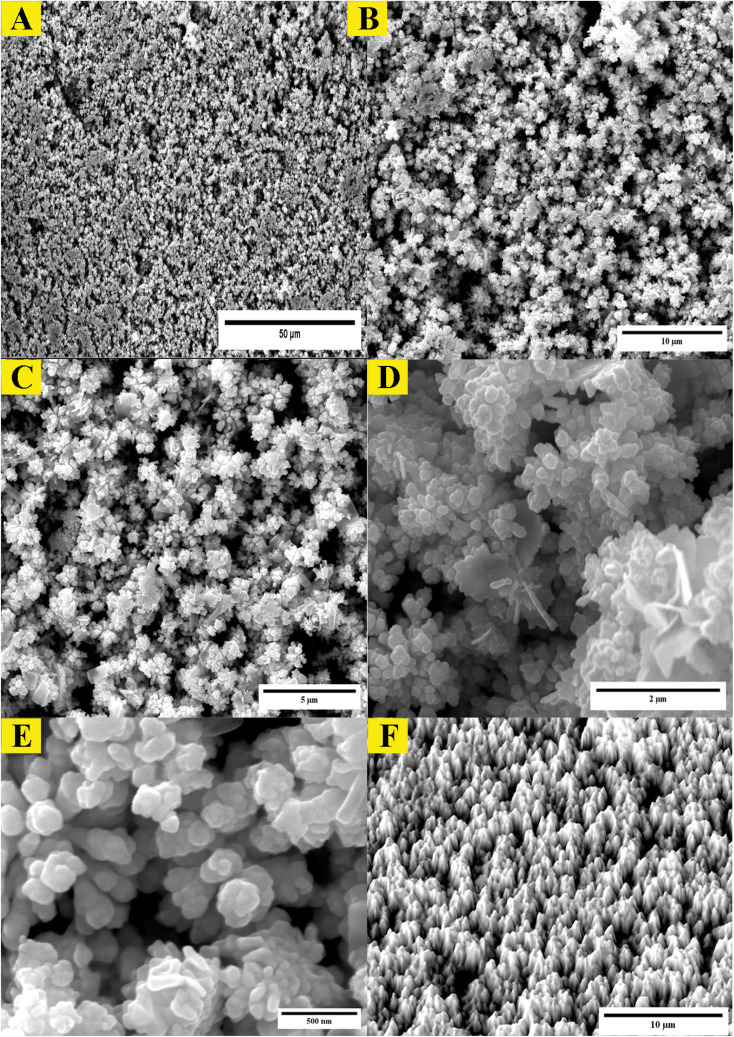
Morphological analysis of synthesized mesoporous AuNPs sponges. (A–E), the FE-SEM images of synthesized AuNPs using the optimum conditions. (F) The 3D surface morphology analysis of AuNPs obtained from ImageJ software.

Examination of the synthesized AuNPs using field FE-SEM confirmed the effectiveness of the synthesis method in producing AuNPs that displayed a distinctive mesoporous sponge-like architecture. Morphological characterization of the AuNPs *via* FE-SEM elucidated key physical attributes. FE-SEM images revealed the nanoparticles were nearly spherical with an average diameter of 10–20 nm. Individual AuNP surfaces appeared smooth and uniform. Moreover, the images confirmed mesoporous material formation within the nanoparticles, seen as lighter regions with pore sizes between 2–5 nm. This FE-SEM analysis therefore offered valuable insights by visualizing the nanoparticles' three-dimensional sponge nanostructure composed of near-spherical AuNPs interspersed with nanoscale pores. Such morphological elucidation using FE-SEM validated the shape and porous composition of the synthesized mesoporous AuNPs. The described morphological properties demonstrated successful synthesis of the targeted nanoparticle system. The TEM micrographs in Fig. 3A–D clearly demonstrate the formation of a highly porous, three-dimensional network structure comprised of mesoporous spherical gold nanoparticles approximately 10–20 nm in size self-assembled under the reaction conditions defined as optimum. The network manifests regular interconnectivity between the nanoparticles, creating an interconnected porous architecture visualized as slightly darker regions between the nanoparticles. This characterization offers compelling evidence that the synthesis yielded the desired mesoporous gold nanoparticle sponges. The energy-dispersive X-ray spectroscopy (EDX) profile presented in Fig. 3E helps substantiate the chemical composition analysis. The strong gold signal along with minor peaks for carbon and copper verify the predominant presence of gold, while also indicating residual compounds stemming from reagents and/or the TEM grid. Overall, the morphological evaluation through TEM imaging and EDX spectroscopy corroborates the successful synthesis of three-dimensional, mesoporous AuNP sponges with the intended nanoscale features and nanostructure using the optimized conditions reported. Remarkably, the sponge structures demonstrated excellent stability under normal environmental conditions for a minimum of 4 months, remained largely unchanged even after vigorous sonication. Thus, this novel approach holds great promise for the scalable production of Au sponges *via* developed colorimetric method (Fig. 4).

**Fig. 5:**
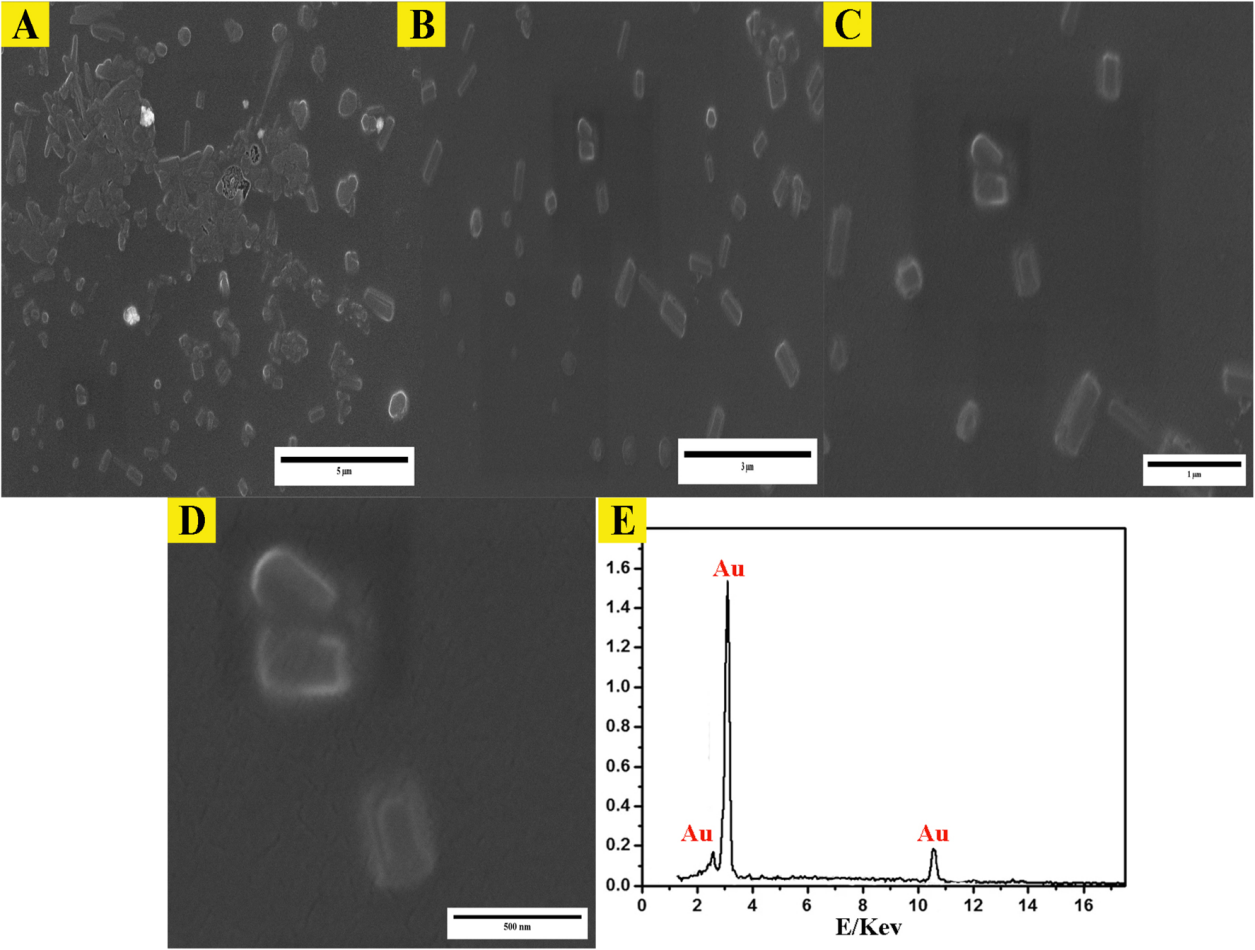
Morphological analysis of synthesized mesoporous AuNPs sponges. (A–D) the TEM images of synthesized AuNPs using the optimum conditions. (E) The EDX of mesoporous of synthesized AuNPs sponges.

### 3.3 Quantitation and control experiments of CPZ

**Fig. 6:**
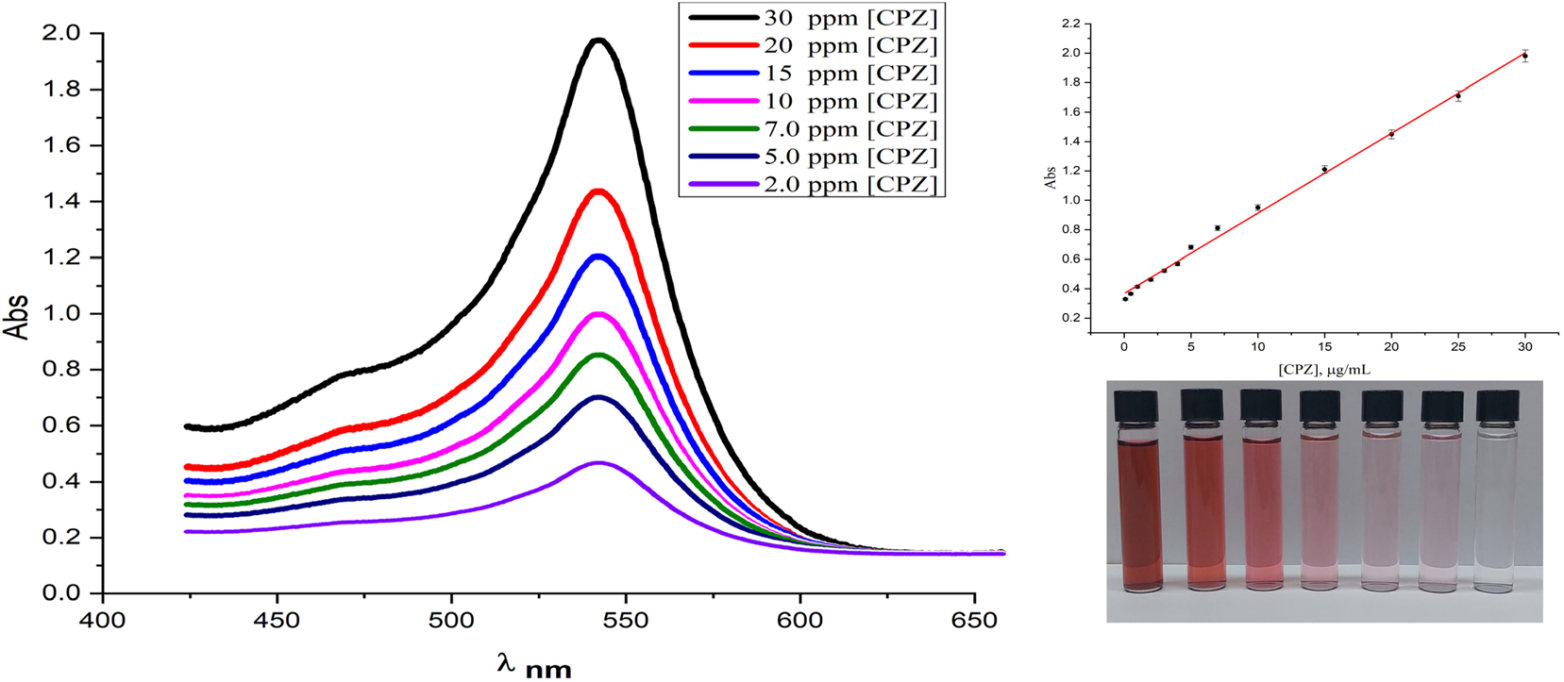
The corresponding UV-vis spectra of AuNPs in serum samples using the optimal conditions with varied CPZ concentrations (2, 5, 7, 10, 15, 20 and 30 μg mL^−1^). Inset: some of visual color analysis of AuNPs and the calibration curve of the absorbance of AuNPs at 527 nm *vs.* CPZ concentration.

The present study reports on the quantitation and control experiments of chlorpromazine (CPZ) using the SPR absorption of AuNPs at 527 nm. The absorbance was found to be directly proportional to the concentration of CPZ, as demonstrated in Fig. 6, which shows the UV-vis spectra of AuNPs in the presence of varying concentrations of CPZ. The linearity between the absorbance at 527 nm and CPZ concentration was observed within the range of 0.1 μg mL^−1^ to 30 μg mL^−1^, as indicated in Fig. 6 (inset), thereby validating the feasibility of quantitative detection of CPZ in solutions utilizing this method. Furthermore, the linear range, limit of detection (LOD), and limit of quantitation (LOQ) of CPZ were determined and summarized in ESI (Table 1S†). Moreover, the operational stability of the proposed assay was assessed by subjecting standard solutions containing 10 and 20 μg mL^−1^ of chlorpromazine (CPZ) to the colourimetric method. The absorbance responses of the 10 and 20 μg mL^−1^ CPZ standards were then measured and compared to the initial absorbance value obtained for the calibration curve. It is noteworthy that only a 4% reduction in absorbance was observed for both the 10 and 20 μg mL^−1^ CPZ standards relative to the initial calibration curve absorbance values, indicating good operational stability of the assay over multiple analyses.

The repeatability and reproducibility of the colorimetric assay were assessed by performing replicate analyses of quality control (QC) samples. To determine repeatability, six successive measurements of 10 μg mL^−1^ and 20 μg mL^−1^ CPZ QC standards were conducted using the established colorimetric method. The relative standard deviation (RSD%) was calculated from the absorbance values to quantify the repeatability of the assay, yielding an RSD of 2.65%. Reproducibility was evaluated on different days by performing six independent colorimetric analyses each of 10 μg mL^−1^ and 20 μg mL^−1^ CPZ QC standards. The RSD was calculated from the absorbance readings to quantify the assay reproducibility between analyses. An RSD value of approximately 3.1% was obtained, as reported in ESI Table S2.† In general, some of the most common interferences in serum for drug detection include endogenous substances such as proteins, lipids, and metabolites that may interfere with the accuracy of results. In addition, exogenous substances such as dietary supplements and medications may have cross-reactivity with the drug being tested for, leading to false-positive or false-negative results. Contamination during various stages of sample handling for drug testing can undermine result accuracy and reliability. Adhering to stringent protocols and validated analytical techniques is crucial to minimize interference and ensure quality outputs. As such, a study tested certain compounds that might falsely influence detection of the target analyte. Absorbance data for AuNP solutions with diverse potential interferents (presented in Fig. 1S of the ESI†) showed only chlorpromazine (CPZ) induced a visible color change in the reaction mixture, raising absorbance at 527 nm. The solutions were exposed to methanol, ethanol, creatinine, uric acid, urea, xanthine, catechol, lactose, sucrose, glucose, fructose and CPZ, but only CPZ elicited a discernible colorimetric reaction. This demonstrated selective AuNP detection of CPZ over an array of possible confounders. Proper experimental controls help verify test selectivity towards the analyte independent of inadvertent effects from co-analytes. On the other hand, the other tested compounds did not cause any significant changes in absorbance at this wavelength, and the reaction solution remained colorless, indicating the absence of AuNPs. Given its reductive properties, ascorbic acid was also evaluated as a potential interferent. The investigation revealed that ascorbic acid would interfere with CPZ detection, but only at extremely high concentrations exceeding 5000 μg mL^−1^. Below this level, ascorbic acid did not elicit a colorimetric response from the AuNP system or falsely influence quantification of CPZ. This threshold level suggests ascorbic acid is an unlikely confounder under normal physiological conditions. Nonetheless, establishing its limit of non-interference helped further validate the selectivity and accuracy of the assay for CPZ determination in real-world samples containing low ascorbic acid levels. Previous studies have shown that antibiotics like sulfonamides and aminoglycosides are unable to convert NH_4_AuCl_4_ into AuNPs. Therefore, this study demonstrates that the AuNPs assay is suitable for the selective detection of CPZ antipsychotics in liquid samples, even in the presence of common interfering substances. In addition, we examined the impact of relevant compounds to chlorpromazine hydrochloride such as alimemazine, promethazine, methdilazine, oxomemazine and thiethylperazine, varying them with and without an amino group and chloride. Surprisingly, the results demonstrated that none of these compounds caused interference; rather, the solution remained colorless, indicating their inability to facilitate the conversion of the NH_4_AuCl_4_ to gold nanoparticles. This significant finding highlights the specific and selective nature of chlorpromazine hydrochloride in the synthesis of gold nanoparticles, reaffirming its potential as a promising agent in nanomaterial synthesis applications.

### 3.4 The growth mechanism of AuNPs

**Fig. 7:**
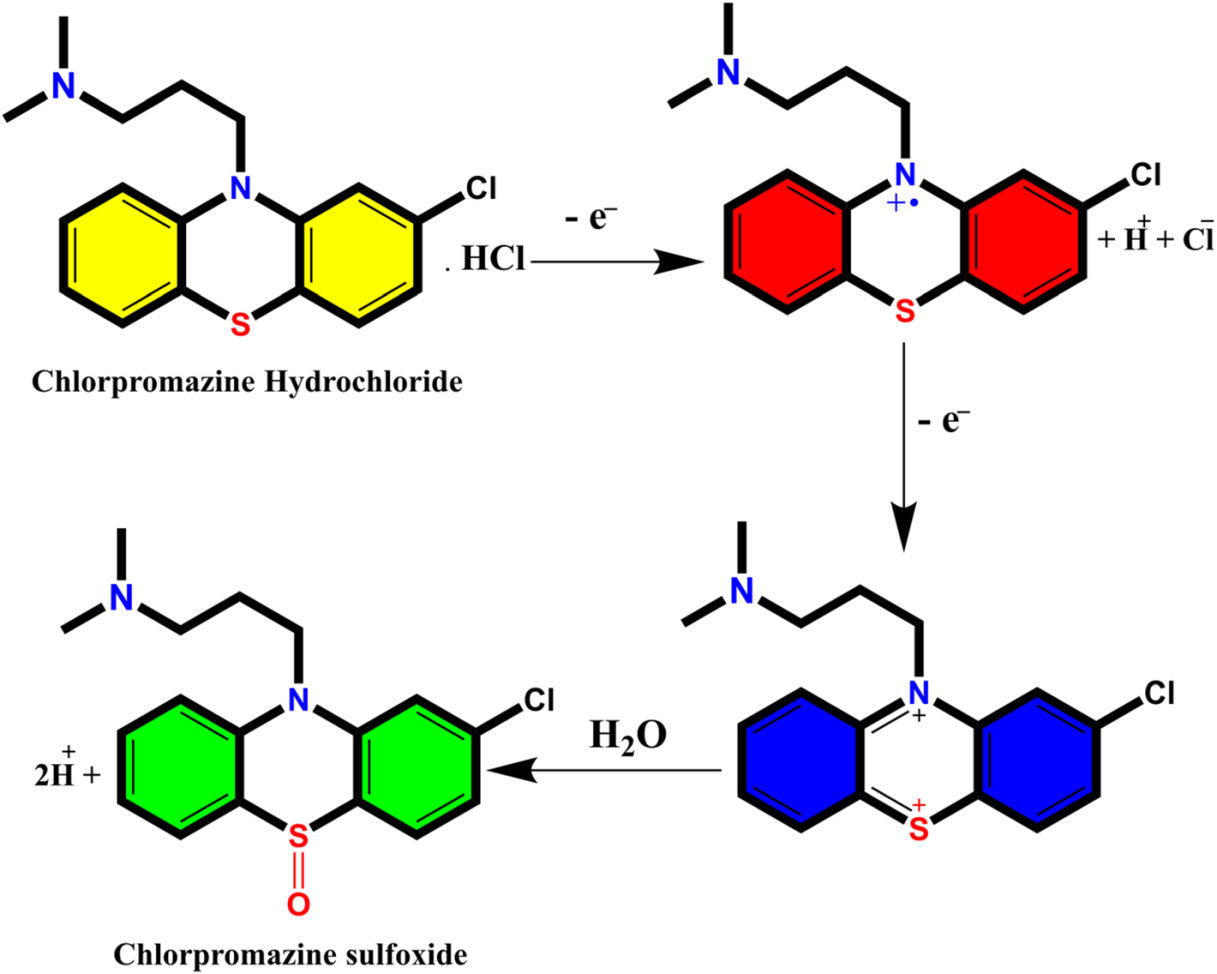
Mechanism oxidation of CPZ.

The investigation of AuNPs growth mechanisms has provided interesting insights into the reduction processes of NH_4_AuCl_4_. Previous studies have shown that molecules with amino groups can directly reduce many of gold salts such as HAuCl_4_·4H_2_O into AuNPs,^35,36^ even at room temperature, without the need for a mediator or seed NPs. This reduction process occurs when the amino groups reduce HAuCl_4_·4H_2_O through H^+^ transfer, resulting in the formation of AuNPs. Recent findings suggest that β-agonists with mono phenolic groups can also reduce HAuCl_4_·4H_2_O into AuNPs, but at higher temperatures.^37^ This indicates that their reducing ability may not be as strong as that of amine compounds. The complex reaction mechanism involving chlorpromazine (CPZ) and NH_4_AuCl_4_ can be further explained by the oxidation of phenothiazine derivatives under neutral conditions. This process involves removing one electron from the nitrogen atom of the ring, leading to the formation of a stable cation radical. Removing another electron generates a phenazothiazonium ion, ultimately resulting in the formation of promethazine sulfoxide (Fig. 7). It is through this process that the reduction of NH_4_AuCl_4_ into AuNPs can occur. The mechanism of CPZ oxidation is thoroughly elucidated, providing a comprehensive understanding of the process. By drawing upon previous works that have already explored and documented the mechanism, this study solidifies its findings and reinforces the credibility of the research.^38–41^ The implications of these findings are significant for the development of new methods for nanoparticle synthesis and could lead to exciting new discoveries in the field. Since, in our experiment, the quantity of gold sponge is 0.215 g by 0.5 g NH_4_AuCl_4_, and the conversion is about 95.13%. Furthermore, a comprehensive investigation was conducted to explore the formation and morphology of these mesoporous gold sponges.

**Fig. 8:**
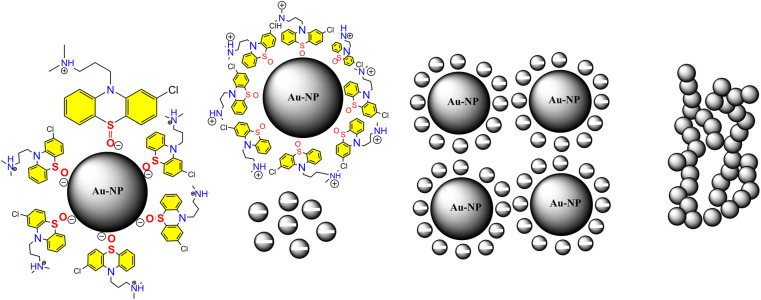
Shows growth mechanism of the synthesized mesoporous AuNPs sponges using the developed colorimetric assay.

The process starts with amine of CPZ (Step 1), which promotes the rapid reduction of NH_4_AuCl_4_, resulting in the formation of small gold particles within the growth solution. These small particles carry a negative charge, which makes them infiltrate and bind to the CPZH^+^, which act as a stabilizer agent to Au seeds (Step 2). The CPZH^+^-stabilized Au seeds act as templates and selectively adhere to these Au seeds, thereby reducing the surface energy of these planes. The CPZ in the solution acts as a capping agent, forming a protective shell around on the surface of the Au seeds. As a result, the small negatively charged Au particles bind to the CPZ-stabilized Au seeds through electrostatic adsorption, and the chain begins to appear (Step 3) (Fig. 8). This mechanism offers an explanation for the phenomenon observed in the TEM image (Fig. 5C and D). The dissolution of smaller seeds into individual atoms and the subsequent growth of larger chains at their expense transpires *via* an Ostwald ripening process, facilitated by continuous reflux. This phenomenon can be attributed to the discrepancy in surface energy between the larger and smaller gold nanoparticles. The gold chains gradually transform into rod-shaped structures, eventually leading to the formation of three-dimensional mesoporous gold sponge architectures. Therefore, the formation process of mesoporous gold sponge at different reaction times can be comprehended as an uninterrupted sequence involving four steps: first: nucleation, second: adsorption, third: growth, and finally, branching. The mechanism of AuNP growth has been thoroughly investigated, and the findings exhibit strong concurrence with the SEM, EDX, and TEM images obtained in our study. These results provide compelling validation for our research methodology and outcomes.^42–44^

### 3.5 Detection of CPZ in real samples

In order to evaluate the practicality of the recommended colorimetric assay, an analysis of human serum and urine samples was conducted using a newly developed colorimetric method. The serum and urine samples were obtained from local hospitals in Iraq, additionally, a CPZ tablet was provided by a local pharmacy in Baghdad, and river water samples were collected at random from various locations along the Tigris River in Baghdad, Iraq. For the biological samples, 2 mL of each sample was pretreated with TCA method and 50 μL of treated sample was added to 0.5 mL of ammonium chloroaurate salt 20 μg mL^−1^ and diluted with 0.5 mL of 0.05 M phosphate buffer (pH = 7). While the non-biological samples, the same procedure was followed without the TCA step. The diluted samples were then spiked with a known concentration of CPZ (5, 10 and 15 μg mL^−1^) and incubated for 2 minutes at 40 °C, and the resulting absorbance of the colorimetric method was measured. The standard addition method was employed to determine the concentration of CPZ in actual samples, with a linear detection range of 0.1–30 μg mL^−1^ (Fig. 6) and a LOD of 0.06 μg mL^−1^ and LOQ of 0.23 μg mL^−1^. Table 1 presents a comprehensive overview of the comparative analysis conducted between the proposed colorimetric assay and other previously reported methods for CPZ determination. The results clearly demonstrate the enhanced colorimetric assay performance, exceptional limit of detection (LOD), and wide linear range exhibited by our assay when compared to other modified electrodes. The sensitivity of the colorimetric assay using AuNPs was found to be lower in real-world samples compared to neat standard solutions, potentially due to sample matrix interferences; however, sensitivity can be improved with proper sample treatment. The recovery results from the real sample analysis are presented in Table 2, demonstrating the feasibility of the colorimetric method for the determination of CPZ in real samples.

**Table 1:** A comparative evaluation for the determination of CPZ between the developed colorimetric assay with previously reported methods

Method	Matrix	Linear range (μg mL^−1^)	LOD	Reference
Electrochemical	River water	0.003–0.025	9 × 10^−4^	45
Flow injection	Pharmaceutical formulations	1.0–8.0	0.5	46
UHPLC	Urine and serum	0.25–300	0.1	47
UV-spectrophotometry	Pharmaceutical formulations	5–25	3.97	48
Acid–dye	Drugs	20–120	12	49
Fluorescence	Human urine	2.3–31.88	0.01	50
Turbidity	Drugs	0.5–3.0	0.3	51
Colorimetric	Drugs, serum, urine, drugs	0.1–30	0.06	This work

**Table 2:** Quantitative determination of CPZ in real samples using the colorimetric assay

Sample type	Added CPZ (μg mL^−1^)	Found CPZ (μg mL^−1^)	Rec%	RSD^a^%
Human urine	5	4.96	99.20	1.12
	10	9.85	98.50	1.31
	15	14.75	98.33	2.12
Human serum	5	4.78	95.60	1.53
	10	9.65	96.50	1.43
	15	15.12	100.80	2.22
River water	5	4.93	98.60	2.42
	10	10.05	100.50	3.14
	15	14.61	97.40	2.73
CPZ tablet	5	4.67	93.40	2.45
	10	10.05	100.50	1.88
	15	14.98	99.86	1.49

a Relative standard deviation for *n* = 3 (quantifications).

## 4 Conclusion

In conclusion, we have developed a sensitive, low-cost, simple and fast colorimetric assay for detecting chlorpromazine hydrochloride antipsychotic using gold nanoparticles (AuNPs) growth. The test can be easily conducted in the field with minimal equipment, making it suitable for conducting on-site analysis of suspect samples. While our method can detect chlorpromazine hydrochloride antipsychotics in liquid samples, it does not provide specific identification of the antipsychotics or differentiate between different types of antipsychotics. Nevertheless, the colorimetric assay remains effective even when most common interferences or salts are present. We posit that by combining our technique for AuNPs growth with (MIP) molecularly imprinted polymer sample treatment, the specificity of the test can be further enhanced. Ongoing research in our lab is focused on exploring this approach further.

## Ethical statement

The research methodology and protocols described in this study were reviewed and approved by the Research Ethics Committee of the College of Sciences at the University of Baghdad. All experimental procedures involving the collection of human biosamples complied with the ethical standards and guidelines set forth by the approving research ethics board.

## Author contributions

The research was equally conceptualized, investigated, and supervised by Mohammad K. Hammood and Jalal N. Jeber. They jointly designed the methodology and analyzed the research data. The experimental aspects were conducted by Maryam A. Khalaf and Haneen Abdul Hadi Kharaba.

## Conflicts of interest

The authors confirm that there are no relevant financial or non-financial competing interests to report.

## Supplementary Material

RA-014-D3RA05516G-s001
