# Supplementary material for: Rapid colorimetric sensing of chlorpromazine HCl antipsychotic through in situ growth of gold nanoparticles
Source: RSC Adv. 2024 Jan 11;14(4):2327–39. doi: 10.1039/d3ra05516g (PMC10782504; doi:10.1039/d3ra05516g)
Supplement: RA-014-D3RA05516G-s001 [file RA-014-D3RA05516G-s001.pdf]

Electronic Supplementary Material (ESI) for RSC Advances.
This journal is © The Royal Society of Chemistry 2024

Table 1S. The statistical summary data of the linear regression line for the turbidimetric determination of CPZ by the colorimetric assay

**Parameter**	**CPZ**
Linearity (μg mL^{-1})	0.1-30
Regression equation	0.3673±0.00126+0.0547±0.0009x[a]
Slope	0.0547±0.0009
Intercept	0.3673±0.00126
Correlation coefficient, r	0.9983
coefficient of determination, r^2	0.9967
LOD (μg mL^{-1})[b]	0.069
LOQ (μg mL^{-1})[c]	0.23
% RSD [d]	1.52
% E [e]	0.51

[a] The detector signal in mV for the concentration of X μg mL^{-1}
[b] Limit of detection
[c] Limit of quantification
[d] Relative standard deviation percentage for three replicate samples
[e] Error percentage for three replicate samples

Table 2S. The intra and inter-day precision of the developed colorimetric assay.

**[CPZ] µg mL^{-1}**	**Found [CPZ±SD [a]] µg mL^{-1}**	**RSD%**
**Intra-day (*n*=6)**		
10	10.221	1.22
20	30.211	2.65
**Inter-day one (*n*=6)**		
10	10.3143±0.0717	1.81
20	20.522±0.8424	0.93
**Inter-day two (*n*=6)**		
10	10.2141±0.3411	2.54
20	20.7855±0.4262	3.10
**Inter-day three (*n*=6)**		
10	10.2331±0.3315	2.98
20	20.0855±0.2145	1.54

[a] The average of five replicate analyses

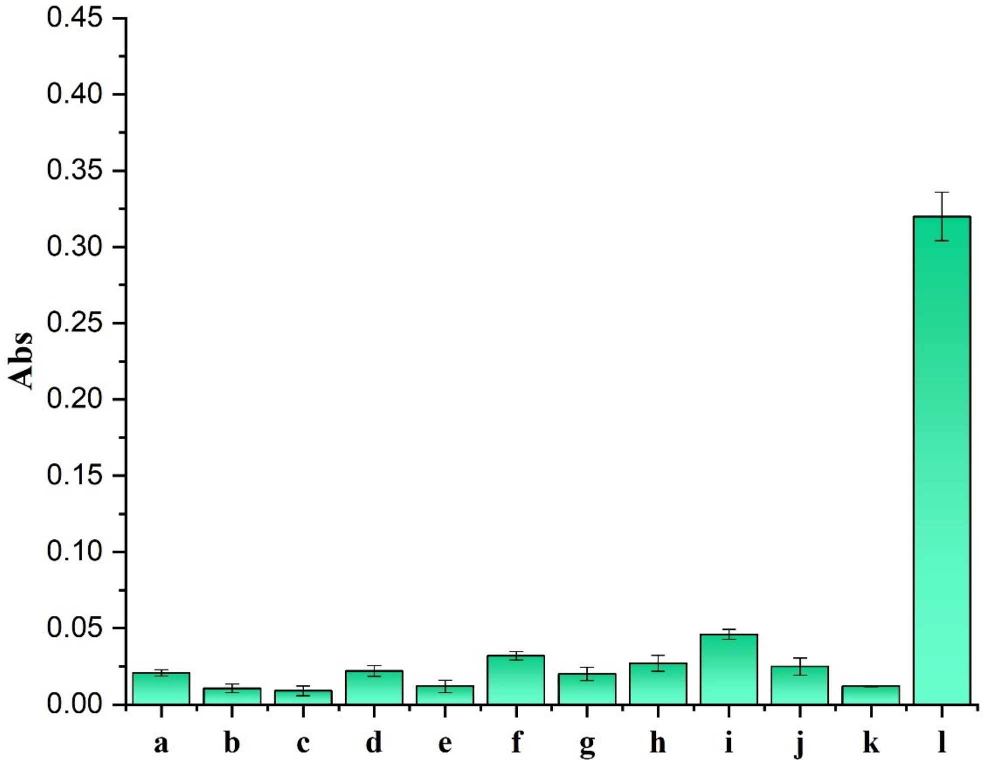

Figure 1S: The absorbance at 527 nm under optimum conditions upon addition the followings: (a) methanol (1:4 v/v), (b) ethanol (1: 4 v/v), (c) creatinine (1000 µg/mL), (d) uric acid (1000 µg/mL), (e) urea (1000 µg/mL), (f) xanthine (1000 µg/mL), (g) catechol (1000 µg/mL), (h) lactose (1000 µg/mL), (i) sucrose (1000 µg/mL), (j), glucose (1000 µg/mL), (k) fructose (1000 µg/mL), (l) ascorbic acid (5000 µg/mL).
